# 

**Published:** 1882-04

**Authors:** 


### *TABLE OF CONTENTS FOR APRIL, 1882.*

ORIGINAL COMMUNICATIONS. PAGE

ART. I.—Laryngeal Phthisis. By J. D. ARNOLD, M. D....................217
ART. II.—The Unnecessary in Medicine and Surgery. By S. V. CLEVENGER, M. D....................223

CLINICAL REPORTS.

Four Cases in the Philadelphia Hospital of Oral Surgery. By JAS. E. GARRETTSON, M. D....................225
CORRECTION....................233

ORIGINAL TRANSLATIONS AND ABSTRACTS.

The Characteristics of Chancres of the Cervix Uteri....................234
Stretching the Optic Nerve....................235
Physiological and Therapeutical Action of Ergot....................236
Treatment of Fracture of the Clavicle by Suturing the Fractured Ends....................237
The Chemistry and Physiological Action of Mercury as used in Amalgam fillings....................238

SELECTIONS.

The Permanent Removal of Hair by Electrolysis.................... 242
Pain in the Foot....................247
Impure Chloroform....................250
Potassium Bromide in Orchitis....................251
What the South Carolina State Board of Health Proposes to Accomplish...252
A New Method of Trephining the Skull....................253
Some Points Concerning Vaccination....................255
Cases of Disease of the Eye from Affections of the Teeth....................256
Carbolized Catgut Sutures for Lacerations of the Cervix Uteri....................258
Dental Surgeons in the Army and Navy....................260
Suppuration without Micro-Organisms....................261
Iodide of Potassium in Frontal Headache....................261
A Triumph of Dentistry....................262
Hysteria....................262
Spontaneous Cure of Vaginal Hernia....................262
Prophylaxis of Venereal Diseases....................263
Deaths from Chloroform....................264
Political Myopia....................265
Maternal Impressions....................265

EDITORIAL DEPARTMENT.

A Chapter on Medical Ethics....................266
Dental Department of University of Maryland....................269
CURRENT NEWS AND OPINION....................270–272
REVIEWS AND BIBLIOGRAPHICAL NOTICES....................272–275

POPULAR SCIENCE DEPARTMENT.

Birth and Breeding....................276
Soda in Burns and Scalds....................282
Monkeys....................283
Babocns....................284
Plant Longevity....................286
Solar Energy....................287
Chemical Difference Between Living and Dead Protoplasm....................287

